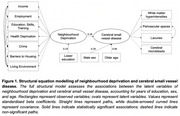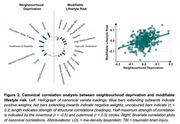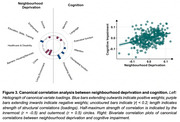# Disadvantaged neighbourhoods, modifiable risk factors, and cerebral small vessel disease in healthy midlife adults: the PREVENT Dementia study

**DOI:** 10.1002/alz70860_106158

**Published:** 2025-12-23

**Authors:** Audrey Low, Georgios Ntailianis, Kamen A Tsvetanov, Maria A Prats‐Sedano, Elizabeth Frances McKiernan, Stephen F Carter, James D Stefaniak, Stefania Nannoni, Anna McKeever, Li Su, Maria‐Eleni Dounavi, Graciela Muniz‐Terrera, Katie Bridgeman, Sarah Gregory, Karen Ritchie, Brian A Lawlor, Lorina Naci, Paresh Malhotra, Ivan Koychev, Craig Ritchie, John T O'Brien, the PREVENT Dementia Investigators

**Affiliations:** ^1^ Mayo Clinic, Rochester, MN, USA; ^2^ Edinburgh Dementia Prevention, University of Edinburgh, Edinburgh, United Kingdom; ^3^ Department of Clinical Neurosciences, University of Cambridge, Cambridge, Cambridgeshire, United Kingdom; ^4^ University of Cambridge, Cambridge, Cambridgeshire, United Kingdom; ^5^ University of Sheffield, Sheffield, United Kingdom; ^6^ Department of Social Medicine, Ohio University, Athens, OH, USA; ^7^ Scottish Brain Sciences, Edinburgh, Scotland, United Kingdom; ^8^ INSERM, Montpellier, France; ^9^ Trinity College Dublin, Dublin, Ireland; ^10^ Imperial College London, London, United Kingdom; ^11^ University of Oxford, Oxford, United Kingdom; ^12^ University of St Andrews, St Andrews, Scotland, United Kingdom; ^13^ Cambridgeshire and Peterborough NHS Foundation Trust, Cambridge, United Kingdom

## Abstract

**Background:**

Individuals living in socioeconomically disadvantaged areas are disproportionately affected by dementia. However, the pathway leading from neighbourhood deprivation to cognitive symptoms is not well understood. To test our hypothesis that this relationship is associated with cerebral small vessel disease (SVD), we examined (1) whether neighbourhood deprivation related to midlife SVD burden and cognition, and (2) whether these links can be explained by modifiable lifestyle risk factors.

**Method:**

In this multi‐centre cross‐sectional study, 514 cognitively healthy midlife participants aged 40‐59 years (median 52 years, 64.6% female) underwent clinical assessment and 3T MRI. Postcode data were used to obtain national indices of neighbourhood deprivation. To quantify SVD, we assessed white matter hyperintensities (WMH), perivascular spaces, cerebral microbleeds, and lacunes. Cognition was assessed using the Computerized Assessment of Information Processing (COGNITO) battery. Lifestyle risk factors were evaluated based on clinical data. Using multivariate statistics like structural equation modelling (SEM) and canonical correlation analysis (CCA), we examined associations between these constructs both globally and at the item‐level (i.e., distinction between domains of cognition/deprivation), to shed light on specific domains that could inform targeted prevention strategies.

**Result:**

Neighbourhood deprivation related to greater prevalence of lifestyle risk factors (*r* = 0.36, *p* < .001), greater SVD burden (b=0.18, *p* = .01; Figure 1), and greater cognitive impairment (*r* = 0.36, *p* < .001), independent of educational attainment, sex, and age. These links with neighbourhood deprivation were largely driven by lifestyle factors relating to vascular health (sleep, physical activity, obesity, hypertension) (Figure 2), and cognitive deficits consistent with SVD (processing speed, visuospatial) (Figure 3). Residents of deprived neighbourhoods displayed greater prevalence of lifestyle risk factors, except alcohol consumption. Lower cognitive scores were most closely associated with deprivation domains of Crime and Living Environment (Figure 3). The *DEPRIVATION→SVD* path was mediated by lifestyle risk factors (z=2.57, *p* = .010), and the *DEPRIVATION→COGNITION* path was mediated by SVD (z=‐2.14, *p* = .032) (global SVD & hypertensive subtype, but not CAA‐SVD).

**Conclusion:**

The pathway linking neighbourhood disadvantage to cognitive impairment at midlife is influenced by vascular risk factors and cerebrovascular burden. Tailored strategies could promote resilience against dementia by promoting health behaviours aligned with the community's unique needs.